# A Case of Cervical Squamous Cell Carcinoma Developing 33 Years After Conization

**DOI:** 10.7759/cureus.52271

**Published:** 2024-01-14

**Authors:** Kayo Inoue, Hiroshi Tsubamoto, Sachiyo Narita, Makoto Yoshida, Kanako Yoshiyasu

**Affiliations:** 1 Department of Obstetrics and Gynecology, Konan Medical Center, Kobe, JPN; 2 Department of Obstetrics and Gynecology, School of Medicine, Hyogo Medical University, Nishinomiya, JPN; 3 Department of Surgical Pathology, School of Medicine, Hyogo Medical University, Nishinomiya, JPN

**Keywords:** peritoneal dissemination, laparoscopy, hematometra, conization, cervical cancer

## Abstract

We report a fatal case of early postoperative peritoneal dissemination in a patient who was diagnosed with cervical squamous cell carcinoma after laparoscopic hysterectomy for hematometra.

A 73-year-old multiparous woman with pyometra and lower abdominal pain was referred to our hospital. Her medical history was remarkable for four open surgeries and conization at the age of 40 years. The cytology obtained from the mucosa of the palpated cervix was negative. The cytology and bacterial culture of the mucus collected from the uterine cavity were negative. Increasing fluid accumulation in the uterine cavity started to cause severe abdominal pain. A laparoscopy was performed. The small intestine showed extensive adhesions to the abdominal wall, which were dissected. A total hysterectomy was performed, and the uterus was placed in a collection bag, cut inside the bag, and retrieved transvaginally. Histopathological examination revealed nests of squamous cell carcinoma that replaced the entire uterine myometrium, and the tumor cells showed diffuse positivity for p16 on immunostaining. The patient was diagnosed with squamous cell carcinoma of the uterine cervix with invasion of the uterine myometrium. Three months later, the patient suffered from small bowel obstruction. A laparotomy was performed, and it revealed numerous disseminated lesions in the pelvic peritoneum and mesentery of the small intestine. Bypass surgery was performed. A biopsy of a disseminated lesion near the vaginal cuff revealed squamous cell carcinoma. The patient died within three weeks of bypass surgery.

## Introduction

Hematometra can be caused by cervical or endometrial cancer, and a preoperative diagnosis may be difficult. Conization can also cause cervical stenosis leading to hematometra. There is a risk of developing cervical cancer late after conization for cervical intraepithelial neoplasia [1,2]. Laparoscopic surgery for cervical cancer carries the risk of peritoneal dissemination due to tumor exposure [3]. Herein, we report a fatal case of early postoperative peritoneal dissemination in a patient who was diagnosed with cervical squamous cell carcinoma after laparoscopic hysterectomy for hematometra. The patient had undergone conization more than 30 years back.

## Case presentation

A 73-year-old multiparous woman with pyometra and lower abdominal pain was referred to our hospital. Her medical history was remarkable for four open surgeries: appendectomy; bilateral salpingectomy due to fallopian tube adhesions; bilateral oophorectomy for ovarian hemorrhage resulting in menopause in her 30s; and intestinal adhesiolysis. In addition, she underwent conization for cervical “high-grade dysplasia” at the age of 40 years. The final pathological result was high-grade dysplasia, which is equivalent to the current high-grade squamous intraepithelial lesion (HSIL)/cervical intraepithelial neoplasia 3 (CIN3), with negative margins. Human papillomavirus (HPV) testing was not performed at the time. The patient was followed up for five years after conization, during which cervical cytology was negative, and after the last visit, no gynecological observation was performed. The patient had no sexual activity after the conization procedure.

At the time of admission, on gynecological examination, the cervix was not visible but was palpable. The cytology obtained from the mucosa of the palpated cervix was negative for intraepithelial lesion or malignancy (NILM). HPV testing was not performed. Transvaginal ultrasonography revealed an enlarged uterine cavity without protruded endometrial lesions. Brown-colored odor-free mucus in the uterine cavity was collected transvaginally using a follicle puncture needle, and both cytology and bacterial culture of the mucus were negative. Serum levels of squamous cell carcinoma antigen (SCC), carcinoembryonic antigen (CEA), and cancer antigen 125 (CA125) were within the normal range (1.4 ng/mL, 2.7 ng/mL, and 11.8 U/mL, respectively).

**Figure 1:**
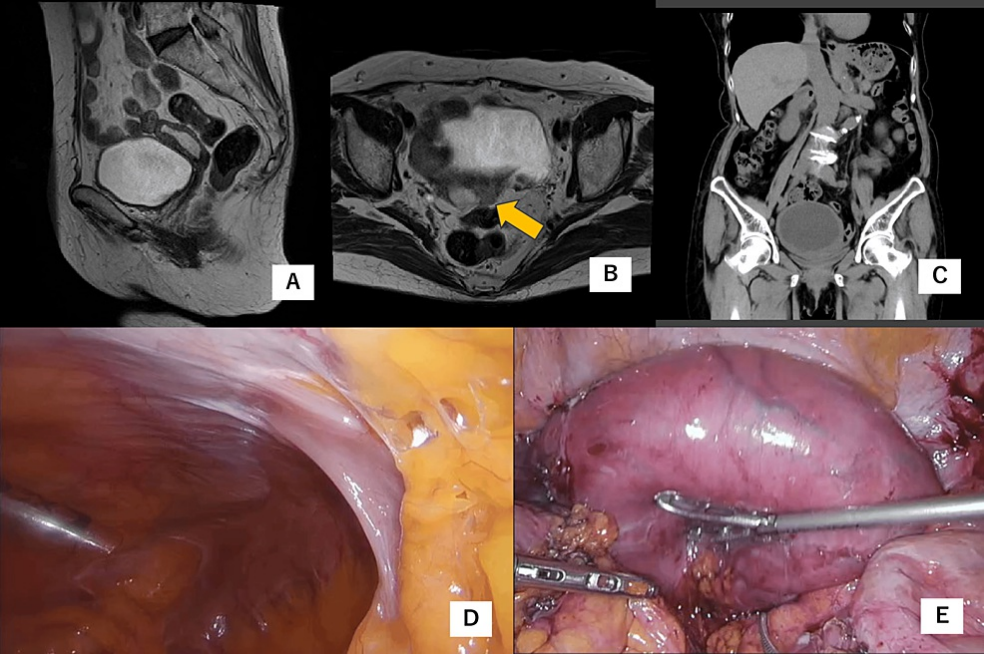
Preoperative imaging studies and laparoscopic surgery (A and B) Magnetic resonance image (T2 image) acquired after drainage. (A) A small amount of fluid was retained in the uterine cavity. (B) The arrow shows a lesion on the left wall of the uterine body with a pale high-signal. This lesion is high-signal on diffuse-weighted imaging and its apparent diffusion coefficient is low (not shown). (C) Computed tomography performed on the day before surgery. The uterus was enlarged with hematometra. (D and E) Laparoscopic surgery. (D) The small intestine was adherent to the abdominal wound. (E) Enlarged uterus. Pelvic peritoneal dissemination was not observed.

Pelvic magnetic resonance imaging (MRI) after puncture drainage revealed a small amount of fluid retention in the uterine cavity (Figure 1A) and a 1 cm lesion on the left wall of the uterine myometrium suspicious of uterine malignancy (Figure 1B). The patient did not agree to undergo a hysterectomy because there were no definitive findings of malignancy, and multiple surgeries had traumatized her to postoperative adhesions.

**Figure 2:**
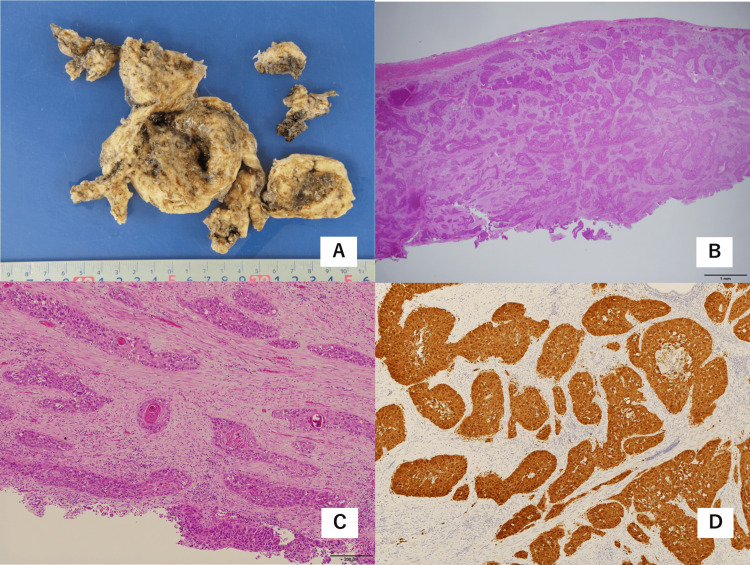
Macroscopic and microscopic findings of the uterus (A) Macroscopic findings of the morcellated pieces of the uterine body after formalin fixation. (B) Hematoxylin and eosin (H&E) staining, magnification ×1.25. Nests of squamous cell carcinoma in the uterine myometrium. There was no apparent endometrium (at the bottom side of the figure). (C) H&E staining, magnification ×10. Squamous cell carcinoma, non-keratinizing type. (D) Immunohistochemical staining for p16. Diffuse nuclear and cytoplasmic staining of squamous cell carcinoma.

The patient remained asymptomatic for eight months. However, increasing fluid accumulation in the uterine cavity, as shown by CT examination (Figure 1C), caused abdominal pain. Oral nonsteroidal anti-inflammatory drugs (NSAIDs) did not relieve the pain. The patient finally agreed to undergo surgery. A laparoscopy was performed. The small intestine showed extensive adhesions to the abdominal wall (Figure 1D), which were dissected. Because of the dense adhesions of the small intestine and rectum to the uterus (Figure 1E), the cervical opening was dilated, and brown-red fluid was drained from the uterine cavity to obtain a better surgical view. No pelvic peritoneal dissemination was observed. A total hysterectomy was performed. After intraperitoneal colpotomy between the uterus and the vagina, the uterus was placed in a collection bag, cut inside the bag, and retrieved transvaginally. The uterine corpus was fragile with no macroscopic lesions in the endometrium, and the cervix was not visible (Figure 2A). Histopathological examination revealed nests of squamous cell carcinoma that replaced the full layer of the entire uterine myometrium without the involvement of the serosa (Figures 2B, 2C). No cervical glands were microscopically identified, and the distal margin failed to be evaluated because the orientation of the morcellated specimen was unclear. No endometrium was microscopically observed. It was probable that long-term inflammation in the uterine cavity had peeled off the endometrium. Histopathology showed no endometrial involvement, and the tumor cells showed diffuse positivity for p16 on immunostaining (Figure 2D). The patient had a history of conization. Therefore, the patient was finally diagnosed with squamous cell carcinoma of the uterine cervix with invasion of the uterine myometrium.

Postoperative concurrent chemoradiotherapy was considered; however, the patient refused since its side effects could reduce her quality of life. The patient did not agree to undergo further imaging studies to identify metastasis. The cytology of the vaginal cuff obtained one month after hysterectomy was NILM.

**Figure 3:**
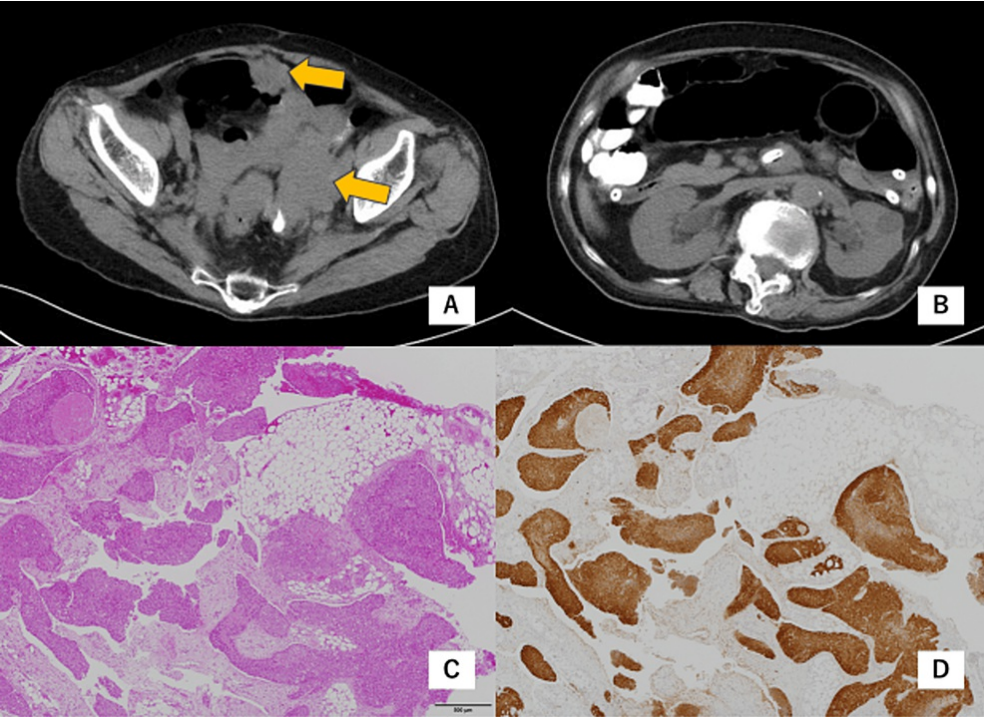
Computed tomography and microscopic findings of the disseminated lesions (A and B) Computed tomography performed on the day of the bypass surgery. (A) The arrows show tumors. (B) Small bowel obstruction and bilateral hydronephrosis. (C) H&E staining of the biopsied tumors, magnification ×4. Squamous cell carcinoma. (D) Immunohistochemical staining of p16 in the biopsied tumor.

Three months later, the patient suffered from intense generalized itching and abdominal pain. CT showed small bowel obstruction. The placement of an ileus tube did not improve her condition in two weeks and the patient worsened. CT showed many mesenteric masses (Figure 3A) that had not been detected on the CT performed two weeks earlier. Bilateral hydronephrosis and lower ureteral stenosis were suspected (Figure 3B). A laparotomy was performed and it revealed numerous disseminated lesions in the pelvic peritoneum and mesentery of the small intestine, which caused intestinal obstruction. Bypass surgery was performed. A biopsy of a disseminated lesion near the vaginal cuff revealed squamous cell carcinoma (Figures 3C, 3D). Her renal function and general condition rapidly deteriorated, and she died within three weeks of bypass surgery.

## Discussion

This case report provides two novel insights. First, this patient may have had the longest time interval between conization for HSIL/CIN3 and the development of cervical squamous cell carcinoma. It showed an unusual spread of squamous cell carcinoma in the entire myometrial layer of the uterus, and immunostaining for p16 was useful for the diagnosis. Second, tumor spillage and exposure via surgical instruments and circulating carbon dioxide during laparoscopic surgery may have resulted in early fatal recurrence with massive peritoneal dissemination. Although no malignancy was detected in the preoperative cytology and imaging studies, malignancy should have been suspected in patients with a history of conization to avoid tumor exposure during surgery.

This case shows the longest time interval of 34 years between conization and diagnosis of cervical cancer. To the best of our knowledge, there have been no case reports of cervical cancer developing more than 30 years after the conization of CIN3 with negative margins. Sand et al. reported 130,000 patients who underwent conization for CIN3. Of these patients, 10 developed cervical cancer more than 25 years later [1]. McCredie et al. reported that the cumulative percentage of cervical cancer or vaginal vault cancer was 0.7% by 30 years among patients treated adequately for CIN3 and mentioned that invasive cancer occurred after 32 years in one patient [2].

Endometrial squamous cell carcinoma may be caused by cervical stenosis, pyometra, and chronic inflammation [4]. In our case, carcinoma of the uterus and that of the cervix had to be differentiated. The removed uterus had no gross cervix and no cervical glands were identified microscopically. Squamous cell carcinoma invaded the entire uterine myometrium. No squamous cell carcinoma or adenocarcinoma was observed in the endometrium. HPV-associated squamous cell carcinoma of the cervix invading the uterine myometrium was diagnosed based on the history of conization for CIN3 and diffuse positivity with p16 immunohistochemistry.

Hematometra is associated with cervical stenosis caused by atrophy, radiotherapy, conization, and carcinoma of the cervix or endometrium. Penna et al. showed that conization in postmenopausal patients carries a high risk of cervical stenosis, which could lead to inadequate follow-up of cervical cytology [5]. Moreover, Breckenridge et al. showed that if postmenopausal patients have symptomatic hematometra and cervical stenosis, the chances of cervical or endometrial carcinoma are very high [6]. The cause of hematometra cannot be identified at times even with a dilation and curettage procedure [7]. Malignancy was not diagnosed preoperatively in our patient because there was no endometrial tumor, and no malignant cells were seen in the fluid retained in the uterine cavity. In addition, there was no visible cervix to be biopsied. This case shows that continuous enlargement of hematometra and a history of conization should warn of a potential malignancy, for which a diagnostic hysterectomy might be considered.

Tumor spillage during laparoscopic surgery can result in peritoneal dissemination [3]. In the present case, the originally closed cervix was dilated for drainage to obtain a better surgical view, and the tumor was exposed in the pelvis via colpotomy. Cancer cells might have been exposed to the surgical instruments and circulating carbon dioxide, which may have caused rapid recurrence in the form of multiple peritoneal dissemination. We believe that laparoscopy was necessary for the dissection of adhesions in the small intestine. Before surgery, it was predicted that the small intestine extensively adhered to the old laparotomy wound; therefore, the first trocar was placed in the left upper quadrant to avoid intestinal damage [8]. However, it might have been better to consider the possibility of uterine malignancy and switch to open surgery after laparoscopic adhesiolysis to perform a hysterectomy, which could have prevented tumor exposure, spillage, and subsequent recurrence.

## Conclusions

Increasing hematometra after conization may indicate cervical squamous cell carcinoma. Cervical squamous cell carcinoma can develop more than 30 years after conization. Spillage of cervical cancer during laparoscopic surgery is a potential risk factor for early recurrence. Therefore, the possibility of cervical cancer should be considered during the examination and treatment of hematometra post conization. Laparoscopic procedures that expose tumors should be avoided to prevent dissemination. It should be noted such recurrence could occur in a very short period of time.
